# Analysis of engineering data with an innovative generalization of the Lomax distribution

**DOI:** 10.1371/journal.pone.0334323

**Published:** 2025-10-27

**Authors:** Hibah Alnashri, Hanan Baaqeel, Dawlah Alsulami, Lamya Baharith

**Affiliations:** Department of Statistics, Faculty of Science, King Abdulaziz University, Jeddah, Saudi Arabia; Rafsanjan University of Vali Asr, IRAN, ISLAMIC REPUBLIC OF

## Abstract

As the amount and complexity of engineering data that need to be analyzed and interpreted continue to increase, the development of new distributions with outstanding adaptability is necessary. The aim of this work is to improve the precision of data modeling, particularly with respect to reliability and lifetime analyses. In this regard, a novel distribution called the Lomax Kavya Manoharan exponential (LKME) distribution derived from the exponential form of a hazard rate function is proposed. The introduction of the Kavya Manoharan exponential distribution with the properties of the Lomax distribution promotes the adaptability to capture different patterns of failure rates, thereby providing a better fit for lifetime data. The LKME distribution is highly flexible and accommodates almost all possible forms of densities, including symmetric, skewed, and inverted J-shaped, as well as diverse shapes of the hazard rate function. This ensures its suitability for modeling various applications in engineering and other fields. Monte Carlo simulations are performed to examine the performance of several classical estimation methods according to benchmarks, such as absolute bias and mean squared error. Furthermore, five engineering datasets are analyzed using the novel LKME distribution, which provides a better fit than comparison distributions, as demonstrated by different goodness-of-fit metrics.

## 1 Introduction

Understanding the random and complex behavior in engineering applications and other real-world phenomena through statistical distributions is crucial in data analysis and interpretation. The first essential step to be taken for any statistical analysis is to choose a distribution that precisely models the data, as choosing an incorrect or unsuitable distribution can lead to misleading reliability and risk predictions. As real-world data become increasingly complex, general-purpose distributions with fixed structures can no longer capture this complex behavior, creating the need for more flexible and adaptable distributions.

Recently, several researchers have attempted to construct new families of statistical distributions using strategies meant to generalize classical distributions and improve their fit to complex real data. Following the T-X-transformed family introduced in [1], applying a suitable transformation function to any probability distribution has become a well-known approach for developing new distributions. The T-X-transformed family has contributed to the development of several distribution families, such as the new Weibull-X family proposed by [2], the new Kumaraswamy generalized family of distributions proposed by [3], the new modified Kies family proposed by [4], and the exponential T-X family proposed by [5]. These families, among others, have been used by researchers to develop new distributions. Some examples include the exponentiated odd Lomax exponential distribution proposed by [6], a new extended Gumbel distribution proposed by [7], a new modified Weibull distribution proposed by [8], the Burr XII-moment exponential distribution proposed by [9], the odd Lindley Weibull distribution proposed by [10], and the half logistic-truncated exponential distribution proposed by [11], all of which expand the toolbox for data modeling.

Recently, the Kavya Manoharan (KM) transformation proposed by [12] has been employed in the formation of new distributions. This transformation does not add extra parameters to the base distribution; thus, it is considered a parameter-parsimonious method. Accordingly, various lifetime distributions based on the KM transformation, including the AKM exponential distribution proposed by [13], the KM generalized exponential distribution proposed by [14], the KM inverse length bias distribution proposed by [15], the KM logistic distribution proposed by [16], and the KM Burr X distribution proposed by [17], have been established. Additionally, [12] proposed the KM exponential distribution characterized by the following probability density function (PDF) and cumulative distribution function (CDF):

$$G(t)=\frac{e}{e-1}\left[1-e^{-\left(1-e^{-\beta t}\right)}\right],\;t,\beta >0,$$ (1)

$$g(t)=\frac{\beta e^{-\beta t}e^{e^{-\beta t}}}{e-1}.$$ (2)

Moreover, [18] introduced the Lomax-G family with the following PDF and CDF:

$$F\left(x\right)=\int_{0}^{{\left(\frac{x+\lambda }{\theta }\right)}^{\alpha }}g\left(t\right)dt=G\left\{{\left(\frac{x+\lambda }{\theta }\right)}^{\alpha }\right\},\;x>0,$$ (3)

$$f\left(x\right)=\frac{\alpha }{\theta }{\left(\frac{x+\lambda }{\theta }\right)}^{\alpha -1}g\left[{\left(\frac{x+\lambda }{\theta }\right)}^{\alpha }\right],$$ (4)

where α,θ,λ>0, *λ* is a location parameter, *G*(*t*) and *g*(*t*) represent the CDF and PDF of the baseline distribution, respectively.

In this study, we present a new probability distribution termed Lomax Kavya Manoharan exponential, a new member of the aforementioned Lomax-G family. The introduction of the LKME distribution is motivated by the following considerations:

1. Formulate a novel distribution by extending the KME distribution using the favorable features of the Lomax-G family.
2. Offer a distribution with exceptional data-modeling versatility and accuracy in engineering and several other areas based on lifetime and reliability data analyses.
3. Cover a wide range of density shapes, including symmetric and asymmetric densities, and diverse hazard rate function shapes.
4. Present a robust distribution as an alternative to existing distributions for fitting and modeling different engineering data types.

The remainder of this article is organized as follows. In Sect 2, we introduce the LKME distribution. Some statistical properties of the LKME distribution are described in Sect 3. The parameter estimation methods for the LKME distribution are detailed in Sect 4. Simulations to assess the performance of the estimation methods are presented in Sect 5. The LKME distribution is applied to five real-world datasets in Sect 6 to assess its performance. Sect 7 provides some concluding remarks.

## 2 The Lomax Kavya-Manoharan exponential distribution

In this section, we present the LKME distribution. Its CDF and PDF can be obtained by inserting Eqs (1) and (2) into Eqs (3) and (4) as follows:

$$F(x)=\frac{e}{e-1}\left[1-e^{-\left(1-e^{-\beta {\left(\frac{x+\lambda }{\theta }\right)}^{\alpha }}\right)}\right],\;\alpha ,\beta ,\theta >0,$$ (5)

$$f(x)=\frac{\alpha \beta }{\theta (e-1)}{\left(\frac{x+\lambda }{\theta }\right)}^{\alpha -1}e^{-\beta {\left(\frac{x+\lambda }{\theta }\right)}^{\alpha }}e^{e^{-\beta {\left(\frac{x+\lambda }{\theta }\right)}^{\alpha }}},\;x\geq -\lambda ,$$ (6)

where *λ* is a location parameter.

The survival function, S(x), for the LKME is given by

$$S(x)=1-F(x)=\frac{1}{e-1}\left[e^{e^{-\beta {\left(\frac{x+\lambda }{\theta }\right)}^{\alpha }}}-1\right],$$ (7)

and the hazard rate function, *H*(*x*), is expressed as follows:

$$H(x)=\frac{\left[\frac{\alpha \beta }{\theta }{\left(\frac{x+\lambda }{\theta }\right)}^{\alpha -1}e^{-\beta {\left(\frac{x+\lambda }{\theta }\right)}^{\alpha }}e^{e^{-\beta {\left(\frac{x+\lambda }{\theta }\right)}^{\alpha }}}\right]}{e^{e^{-\beta {\left(\frac{x+\lambda }{\theta }\right)}^{\alpha }}}-1}.$$ (8)

**Fig 1:**
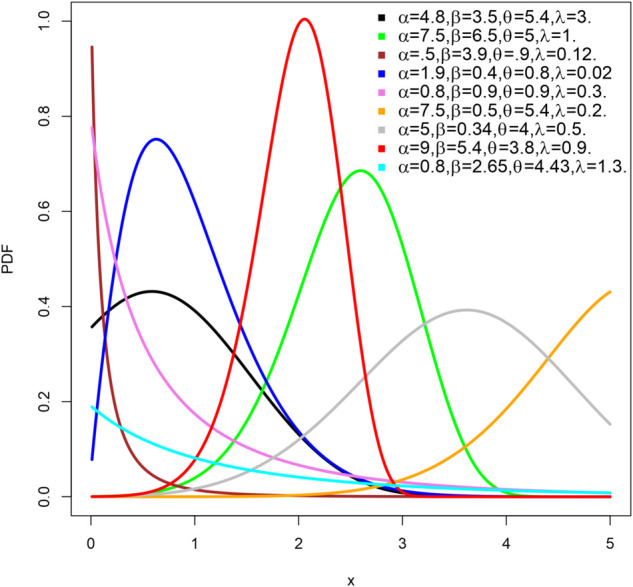
The LKME density plots for some values of the parameters.

**Fig 2:**
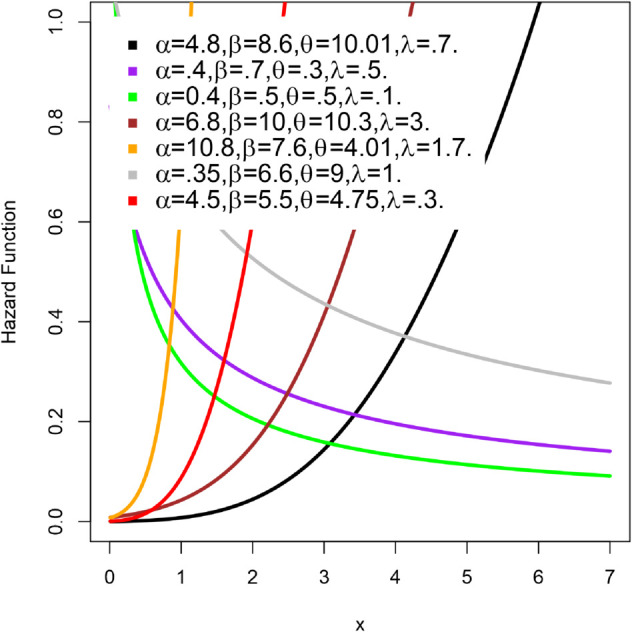
The LKME hazard function plots for some values of the parameters.

The density function and hazard function *H*(*x*) under different parameter settings are shown in Figs 1 and 2, respectively. The shape of the density function can be symmetric, right-skewed, left-skewed or even an inverted J-shape, as seen in Fig 1. Similarly, the hazard function *H*(*x*) in Fig 2 can take various forms: it can increase monotonically, decrease monotonically, be J-shaped, or be reversed J-shaped. These variations highlight the flexibility of the LKME model in effectively capturing a wide range of data distribution behaviors.

### 2.1 Special cases of the LKME distribution

- The Kavya-Manoharan exponential (KME) distribution is obtained when α=θ=1,λ=0 in Eq (6).
- The Kavya-Manoharan Weibull (KMW) distribution is obtained when $\theta =\frac{1}{\theta },\beta =1,\lambda =0$ in Eq (6).
- Kavya-Manoharan Lomax (KM-Lomax) distribution with $\left(\alpha ,\frac{1}{\theta }\right)$ is obtained when θ=λandβ=1 in Eq (6).
- The generalized Kavya-Manoharan exponential (GKME) distribution is obtained by substituting $x=\frac{1}{\theta }{\left\{-\ln\left[1-{\left(1-e^{-\theta y}\right)}^{\alpha }\right]\right\}}^{\frac{1}{\alpha }}$ into Eq (6) and setting $\theta =\frac{1}{\theta },\;\beta =1,\;\lambda =0$.
- The DUS-Lomax distribution is obtained by substituting $x=\frac{1}{\theta }{\left\{-ln\left[ln\left(e+1-e^{1-{\left(1+\theta y\right)}^{-\alpha }}\right)\right]\right\}}^{\frac{-1}{\alpha }}$ in Eq (6) and setting $\theta =\frac{1}{\theta },\;\alpha =-\alpha ,\;\beta =1\;and\;\lambda =0$.

### 2.2 Linear representations of the LKME density

This subsection provides a detailed expansion of the LKME PDF function in (6), Using the following exponential expansion and binomial expansions.

$$e^{z}=\sum_{i=0}^{\infty }\frac{z^{i}}{i!},$$ (9)

(1+z)n=∑j=0n(nj)zj, (10)

results in a linear representation of the LKME density as follows:

f(x)=αβe−1∑i=0∞∑j=0α−1(α−1j)λα−j−1i!θαxje−β(i+1)(x+λθ)α. (11)

## 3 Some statistical properties of LKME

### 3.1 Quantile function

The quantile function of the LKME model can be obtained by inverting the distribution function given in (6).

$$x_{p}=\theta {\left[\frac{-1}{\beta }ln\left[1+ln\left(1-\left(\frac{e-1}{e}\right)p\right)\right]\right]}^{\frac{1}{\alpha }}-\lambda ,$$ (12)

The median can be determined by substituting *p* = 0.5 into Eq (12), as shown below.

$$x_{0.5}=\theta {\left[\frac{-1}{\beta }ln\left[1+ln\left(1-\frac{1}{2}\left(\frac{e-1}{e}\right)\right)\right]\right]}^{\frac{1}{\alpha }}-\lambda .$$ (13)

### 3.2 The Galton skewness and Moors Kurtosis

The Galton skewness (*GS*_*k*_) is a measure of distribution symmetry [19] and is defined as follows:

$$GS_{k}=\frac{x_{0.75}-2x_{0.5}+x_{0.25}}{x_{0.75}-x_{0.5}}.$$ (14)

The Moors kurtosis (MKur) [20], derived from octiles, is defined as follows:

$$MKur=\frac{x_{0.875}-x_{0.625}-x_{0.375}+x_{0.125}}{x_{0.75}-x_{0.5}}.$$ (15)

where *x*_(.)_ denotes the quantile function of the LKME model in Eq (12).

**Fig 3:**
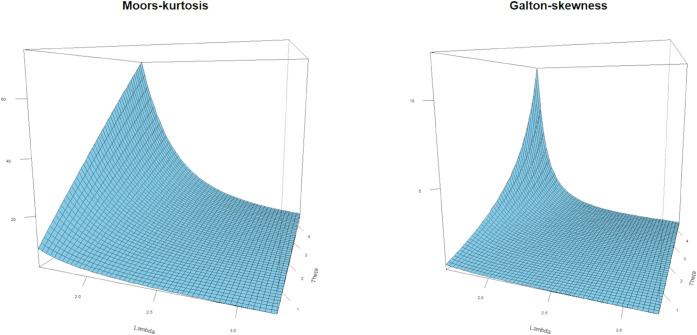
3D visualization of the Moors kurtosis and Galton skewness measures for the LKME distribution.

Fig 3 shows the plots of the Moors kurtosis and Galton skewness as functions of *λ* and *θ*. The Moors kurtosis plot indicates that increasing *λ* and decreasing *θ* result in higher Moors kurtosis and a sharper distribution. In contrast, the Galton skewness plot reveals a strong correlation between Galton skewness and small values *θ*, which are associated with variations in distribution asymmetry. These results illustrate the sensitivity of the factors used as descriptors of the distribution properties.

### 3.3 Moments

If x∼LKME(α,β,θ,λ), then the *r*^*th*^ moment of X can be expressed as follows.


$$\mu_{r}=\int_{-\lambda }^{\infty }x^{r}f(x)dx$$

=αβe−1∑i=0∞∑j=0α−1(α−1j)λα−j−1i!θα∫−λ∞xr+je−β(i+1)(x+λθ)αdx, (16)

By substituting $w\;=\;\beta \left(i+1\right)\;\;{\left(\frac{x+\lambda }{\theta }\right)}^{\alpha }$, we obtain

=βe−1∑i=0∞∑j=0α−1(−1)r+j(α−1j)λα+r−1β(i+1)i!θα−1∫0∞[1−(θλ)(wβ(i+1))1α]r+j(wβ(i+1))1α−1e−wdw. (17)

Next, we use the following series expansion.

(1−z)n=∑k=0n(−1)k(nk)zk. (18)

Thus, the *r*^*th*^ moment of the LKME distribution can be expressed as follows:

μr=βe−1∑i=0∞∑j=0α−1∑k=0r+j(−1)r+j+k(α−1j)(r+jk)λα+r−k−1i!θα−k−1Γ(k+1α)[β(i+1)]k+1α. (19)

Consequently, the mean of the LKME distribution can be expressed as follows:

μ=E(X)=βe−1∑i=0∞∑j=0α−1∑k=0j+1(−1)j+k+1(α−1j)(j+1k)λα−ki!θα−k−1Γ(k+1α)[β(i+1)]k+1α, (20)

Therefore, the variance of the LKME distribution is given by

σ2=E(X2)−μ2=βe−1∑i=0∞∑j=0α−1∑k=0j+2(−1)j+k+2(α−1j)(j+2k)λα−k+1i!θα−k−1Γ(k+1α)[β(i+1)]k+1α−μ2. (21)

### 3.4 Moment generation function

The moment generating function (MGF) of the LKME distribution can be obtained as follows:

$$MGF_{X}(t)=E\left(e^{tX}\right)=\sum_{r=0}^{\infty }\frac{{\left(t\right)}^{r}}{r!}\mu_{r},$$ (22)

Therefore,

MGFX(t)=βe−1∑r=0∞∑i=0∞∑j=0α−1∑k=0r+j(−1)r+j+k(α−1j)(r+jk)λα+r−k−1i!θα−k−1Γ(k+1α)[β(i+1)]k+1α(t)rr!. (23)

### 3.5 Characteristic function

The characteristic function of the LKME distribution is expressed as follows:

$$\phi_{X}\left(t\right)=E\left(e^{itX}\right)=\sum_{r=0}^{\infty }\frac{{\left(it\right)}^{r}}{r!}\mu_{r},$$ (24)

=βe−1∑r=0∞∑i=0∞∑j=0α−1∑k=0r+j(−1)r+j+k(α−1j)(r+jk)λα+r−k−1i!θα−k−1Γ(k+1α)[β(i+1)]k+1α(it)rr!. (25)

### 3.6 Probability weighted moment

The probability weighted moment (PWM) of the LKME distribution is defined as follows:

$$E\left[X^{r}F(X{)}^{s}\right]=\int_{-\infty }^{\infty }x^{r}f(x){\left[F(x)\right]}^{s}dx,$$ (26)

=αβes(e−1)s+1∑i=0∞∑j=0α−1(α−1j)λα−j−1i!θα∫−λ∞xr+je−β(x+λθ)α(i+1)[1−e−(1−e−β(x+λθ)α)]sdx, (27)

The series expansion in Eq (18) and the following exponential series are employed.

$$e^{-z}=\sum_{n=0}^{\infty }\frac{(-1{)}^{n}}{n!}z^{n}.$$ (28)

Thus, we obtain

=αβes(e−1)s+1∑i=0∞∑n=0∞∑j=0α−1∑m=0s∑l=0n(−1)n+m+li!n!(α−1j)(sm)(nl)mnλα−j−1θα∫−λ∞xr+je−β(x+λθ)α(l+i+1)dx, (29)

By substituting $w\;=\;\beta \;{\left(\frac{x+\lambda }{\theta }\right)}^{\alpha }\left(l\;+\;i\;+1\right)$, we obtain the following.

=es(e−1)s+1∑i=0∞∑n=0∞∑j=0α−1∑m=0s∑l=0n(−1)n+m+l+1i!n!(α−1j)(sm)(nl)mnλα+r−1αβθα−1(l+i+1)∫0∞[1−θλ(wβ(l+i+1))1α]r+j[wβ(l+i+1)]1α−1e−wdw, (30)

Using the series expansion in Eq (18), the PWM of the LKME distribution can be expressed as follows:

E[XrF(X)s]=βes(e−1)s+1∑i=0∞∑j=0α−1∑m=0s∑n=0∞∑l=0n∑k=0r+j(−1)n+m+l+k+1(α−1j)(sm)(nl)(r+jk)mni!n!λα+r−k−1θα−k−1Γ(k+1α)[β(l+i+1)]k+1α. (31)

### 3.7 Order statistics

Let $X_{1}<X_{2}<X_{3}<\ldots <X_{n}$ represent a random sample of size *n* drawn from the LKME distribution, where *x*_*r*:*n*_ denotes the *r*^*th*^ order statistic. The PDF of *x*_*r*:*n*_ can be expressed as follows:

fr:n(x)=f(x)β(r,n−r+1)∑v=0n−r(−1)v(n−rv)Fv+r−1(x). (32)

By substituting Eqs (5) and (11) into Eq (32) and then applying the series expansions in Eqs (18) and (28), we obtain the *r*^*th*^ order statistic of the LKME distribution as follows:

fr:n(x)=αββ(r,n−r+1)∑i=0∞∑j=0α−1∑v=0n−r∑k=0v+r+1∑l=0∞∑m=0l(−1)k+l+m+vi!l!(n−rv)(α−1j)(lm)(v+r−1k)λα−j−1klθαev+r−1(e−1)v+rxje−β(m+i+1)(x+λθ)α. (33)

**Proof.** See S1 Appendix

### 3.8 R’enyi entropy

The R’enyi entropy of order *u* is given by

$$RE_{X}(u)=\frac{1}{1-u}log\left(\int_{-\infty }^{\infty }f^{u}(x)dx\right),\;u>0,u\neq 1,$$ (34)

Thus, the R’enyi entropy of the LKME distribution can be expressed as follows:

$$RE_{X}(u)=log\left(\frac{\alpha }{\theta }\right)+\frac{1}{u-1}\left[\left(u-k\right)log\beta -ulog\left(e-1\right)+\sum_{i=0}^{\infty }log\left(\frac{u^{i}}{i!{\left(i+u\right)}^{k}}\right)+\log\left(\Gamma (k)\right)\right].$$ (35)

**Proof.** See S2 Appendix.

### 3.9 Shannon entropy

If a random variable *X* follows the LKME distribution with the PDF given by Eq (6), then the Shannon entropy, *SE*_*X*_, is expressed as follows:

$$SE_{X}=-E\left[log\left(f(x)\right)\right],$$ (36)

Using the following sequence expansions, along with the sequences in Eqs (9) and (18),

$$log(1+z)=\sum_{k=1}^{\infty }\frac{(-1{)}^{k-1}}{k}z^{k}.$$ (37)

Therefore, the Shannon entropy of the LKME distribution is given by

SEX=11−e∑n=0∞∑i=0∞∑k=0i1(n+1)![(ik)(−1)k+1i(α−1)θkλkβkαΓ(kα+1)(n+1)kα−1n+1]+11−e−[log(αβθ(e−1))+(α−1)log(λθ)]. (38)

**Proof.** See S1 Appendix.

## 4 Estimation methods

To evaluate the performance of the LKME distribution, we employed five different estimation techniques, namely, maximum likelihood estimation (MLE), percentile estimation (PE), least squares estimation (LS), weighted least squares estimation (WLS), and the Cramer von Mises method (CVM), to assess the fit of the LKME distribution and accurately quantify its parameters.

### 4.1 Maximum likelihood method

If a random sample $x_{1},x_{2},x_{3},....,x_{n}$ of size *n* is drawn from LKME distribution, then the log-likelihood function (*l*) for the parameters α,λ,θandβ is expressed as follows:

$$l=n\left[log(\alpha )+log(\beta )-log(\theta )-log\left(e-1\right)\right]\;+\sum_{i=1}^{n}\left[(\alpha -1)log\left(\frac{x+\lambda }{\theta }\right)+\beta {\left(\frac{x+\lambda }{\theta }\right)}^{\alpha }+e^{-\beta {\left(\frac{x+\lambda }{\theta }\right)}^{\alpha }}\right],$$ (39)

The MLE of the parameters is obtained by computing the partial derivatives of Eq (39) with respect to each parameter, equating the resultant equations to zero, and solving for the parameters, as follows:

$$\frac{\partial l}{\partial \alpha }=\frac{n}{\alpha }+\sum_{i=1}^{n}\left\{log\left(\frac{x+\lambda }{\theta }\right)+\beta {\left(\frac{x+\lambda }{\theta }\right)}^{\alpha }log(\alpha )\left[1-e^{-\beta {\left(\frac{x+\lambda }{\theta }\right)}^{\alpha }}\right]\right\}.$$ (40)

$$\frac{\partial l}{\partial \beta }=\frac{n}{\beta }+\sum_{i=1}^{n}\left\{{\left(\frac{x+\lambda }{\theta }\right)}^{\alpha }\left[1-e^{-\beta {\left(\frac{x+\lambda }{\theta }\right)}^{\alpha }}\right]\right\}.$$ (41)

$$\frac{\partial l}{\partial \theta }=\frac{-n}{\theta }+\sum_{i=1}^{n}\left\{\frac{1-\alpha }{\theta }-\frac{\alpha \beta }{\theta }{\left(\frac{x+\lambda }{\theta }\right)}^{\alpha }\left[1-e^{-\beta {\left(\frac{x+\lambda }{\theta }\right)}^{\alpha }}\right]\right\}.$$ (42)

$$\frac{\partial l}{\partial \lambda }=\sum_{i=1}^{n}\left\{\frac{\alpha -1}{x+\lambda }+\frac{\alpha \beta }{\theta }{\left(\frac{x+\lambda }{\theta }\right)}^{\alpha -1}\left[1-e^{-\beta {\left(\frac{x+\lambda }{\theta }\right)}^{\alpha }}\right]\right\}.$$ (43)

The system of Eqs (40)–(43) can be solved using some iterative optimization techniques such as R packages [21].

### 4.2 Percentiles method

Suppose that $x_{1},x_{2},x_{3},\ldots ,x_{n}$ is a random sample of size *n* from the LKME distribution. Then, the percentile estimation (PE) method is

$$PE=\sum_{i=1}^{n}{\left[x_{i}-\left(\theta {\left[\frac{-1}{\beta }ln\left[1+ln\left(1-\left(\frac{e-1}{e}\right)p_{i}\right)\right]\right]}^{\frac{1}{\alpha }}-\lambda \right)\right]}^{2},$$ (44)

where *x*_*i*_ is the *i*^*th*^ order statistics of the sample, $p_{i}=\frac{i}{n+1}\;and\;i=1,2,3,....,n.$

The parameter estimates of α,λ,θandβ using the PE method are obtained by minimizing the sum of squares of the difference between the observed sample order statistics and the expected values of the fitted distribution in the corresponding percentiles.

### 4.3 Ordinary least squares estimators

Let $x_{1},x_{2},x_{3},\ldots ,x_{n}$ be a random sample of size *n* from the LKME distribution. The ordinary least squares estimation (LSE) method for estimating the LKME parameters is as follows:

$$LSE=\sum_{i=1}^{n}{\left[F_{LKME}\left(x_{i}\right)-\left(\frac{i}{n+1}\right)\right]}^{2},\;i=1,2,...,n.$$ (45)

That is, the parameters are optimized by minimizing the sum of the squared differences. Thus, the LSE estimator for the unknown parameters of the LKME distribution is obtained by minimizing the following expression.

$$LSE=\sum_{i=1}^{n}{\left[\frac{e}{e-1}\left[1-e^{-\left(1-e^{-\beta {\left(\frac{x_{i}+\lambda }{\theta }\right)}^{\alpha }}\right)}\right]-\left(\frac{i}{n+1}\right)\right]}^{2},\;i=1,2,...,n,$$ (46)

where *x*_*i*_ is the *i*^*th*^ order statistics of the sample.

### 4.4 Weighted least squares estimators

Let $x_{1},x_{2},x_{3},\ldots ,x_{n}$ be a random sample from the LKME distribution. Then, the weighted least squares estimator (WLS) is given by

$$WLS=\sum_{i=1}^{n}\frac{{\left(n+1\right)}^{2}\left(n+2\right)}{i\left(n-i+1\right)}{\left[F_{LKME}\left(x_{i}\right)-\left(\frac{i}{n+1}\right)\right]}^{2}.$$ (47)

The WLS minimizes the following expression to estimate the LKME parameters.

$$WLS=\sum_{i=1}^{n}\frac{{\left(n+1\right)}^{2}\left(n+2\right)}{i\left(n-i+1\right)}{\left[\frac{e}{e-1}\left[1-e^{-\left(1-e^{-\beta {\left(\frac{x_{i}+\lambda }{\theta }\right)}^{\alpha }}\right)}\right]-\left(\frac{i}{n+1}\right)\right]}^{2},$$ (48)

where *x*_*i*_ is the *i*^*th*^ order statistics of the sample.

### 4.5 Cramer-von Mises minimum distance method

The Cramer-von Mises (CVM) method works by minimizing the difference between the estimated CDF and empirical CDF. Specifically, the aim of this method is to determine the parameter values that best align the theoretical distribution with the observed distribution.

$$CVM=\frac{1}{12n}+\sum_{i=1}^{n}{\left[F_{LKME}(x_{i})-\frac{2i-1}{2n}\right]}^{2}.$$ (49)

By incorporating Eq (5) with Eq (49), the CVM estimator for the LKME parameters is obtained by minimizing the following.

$$CVM=\frac{1}{12n}+\sum_{i=1}^{n}{\left[\frac{e}{e-1}\left[1-e^{-\left(1-e^{-\beta {\left(\frac{x_{i}+\lambda }{\theta }\right)}^{\alpha }}\right)}\right]-\frac{2i-1}{2n}\right]}^{2}.$$ (50)

## 5 Simulation study

This section presents the numerical results of a Monte Carlo simulation to assess the performance of five estimation methods: MLE, PE, LSE, WLS, and CVM. Data were generated from the LKME distribution using Eq (12), where *p* was sampled from *Uniform*(0,1). We consider six sample sizes (n=20,50,100,200,300,500) and five sets of parameter values specified as follows:

- Set I: (α=2.51,β=0.82,θ=2.96,λ=0.12).
- Set II: (α=2.85,β=1.47,θ=2.28,λ=0.065).
- Set III: (α=1.7,β=1.96,θ=1.5,λ=0.0035).
- Set IV: (α=2.2,β=1.6,θ=1.95,λ=0.0075).
- Set V: (α=3.7,β=3.87,θ=3.9,λ=0.22).

Parameter estimates are obtained using the “optim” function in R statistical software [21]. In addition, the absolute bias (Bias) and mean squared error (MSE) are calculated as follows:


$$Bias(\hat{\varphi })=\frac{1}{n}\sum_{i=1}^{n}|({\hat{\varphi }}_{i}-\varphi )|,$$

$$MSE(\hat{\varphi })=\frac{1}{n}\sum_{i=1}^{n}({\hat{\varphi }}_{i}-\varphi {)}^{2},$$

where φ is the true parameter value, $\hat{\varphi }$ is the corresponding estimate, and *n* is the sample size.

The average estimates of the parameters based on 1,000 replications using various approaches, with the corresponding |Bias| and MSE, are displayed in Tables 1–5.

**Table 1:** Simulation study at α=2.51, β=0.82, θ=2.96, λ=0.12.

Set I:			MLE			PE			LSE			WLS			CVM	
		Est	Bias	MSE	Est	Bias	MSE	Est	Bias	MSE	Est	Bias	MSE	Est	Bias	MSE
n = 20	*α*	3.5168	1.0068	166.6136	3.0464	0.5364	9.0171	5.1728	2.6628	589.9056	5.6501	3.1401	836.5962	5.9287	3.4187	678.7062
	*β*	1.2347	0.4147	1.4276	0.9386	0.1186	0.6654	1.0990	0.2790	4.3214	1.2041	0.3841	11.3000	1.1738	0.3538	5.5085
	*θ*	3.8457	0.8857	115.5769	3.4392	0.4792	4.9727	5.1207	2.1607	430.6174	5.3619	2.4019	581.4473	5.2542	2.2942	366.7864
	*λ*	0.7718	0.6518	120.1724	0.7018	0.5818	6.7372	2.4179	2.2979	426.3247	2.7137	2.5937	577.5495	2.5984	2.4784	367.4865
n = 50	*α*	2.4870	0.0230	0.4651	2.5485	0.0385	0.9674	2.8138	0.3038	1.6815	3.5929	1.0829	611.6987	3.0326	0.5226	6.3234
	*β*	1.0347	0.2147	0.2642	0.9259	0.1059	0.2561	0.8935	0.0735	0.4662	0.8768	0.0568	0.2793	0.9301	0.1101	0.8082
	*θ*	3.0653	0.1053	0.1537	3.1016	0.1416	0.3958	3.1561	0.1961	0.8012	3.6517	0.6917	263.6617	3.2601	0.3001	4.2065
	*λ*	0.0460	0.0740	0.2368	0.2096	0.0896	0.6151	0.4085	0.2885	1.2134	0.8883	0.7683	263.0649	0.5304	0.4104	4.3612
n = 100	*α*	2.4715	0.0385	0.1472	2.4894	0.0206	0.2484	2.6695	0.1595	0.5771	2.6177	0.1077	0.2843	2.7281	0.2181	0.7477
	*β*	0.9630	0.1430	0.1110	0.8820	0.0620	0.1804	0.8964	0.0764	0.3809	0.8624	0.0424	0.1282	0.8715	0.0515	0.2258
	*θ*	3.0441	0.0841	0.0720	3.0075	0.0475	0.1827	3.0888	0.1288	0.4284	3.0484	0.0884	0.1595	3.0699	0.1099	0.5025
	*λ*	0.0623	0.0577	0.0779	0.1424	0.0224	0.1691	0.2732	0.1532	0.3915	0.2133	0.0933	0.1805	0.2983	0.1783	0.5130
n = 200	*α*	2.4750	0.0350	0.0603	2.4689	0.0411	0.1033	2.6097	0.0997	0.2633	2.5635	0.0535	0.1099	2.6543	0.1443	0.2488
	*β*	0.9119	0.0919	0.0598	0.8656	0.0456	0.0673	0.8528	0.0328	0.0943	0.8643	0.0443	0.0588	0.8549	0.0349	0.1413
	*θ*	3.0195	0.0595	0.0499	2.9869	0.0269	0.1216	3.0459	0.0859	0.2135	3.0373	0.0773	0.1042	3.0499	0.0899	0.2277
	*λ*	0.0816	0.0384	0.0306	0.1094	0.0105	0.0707	0.2151	0.0951	0.1850	0.1639	0.0439	0.0663	0.2385	0.1185	0.1783
n = 300	*α*	2.4856	0.0244	0.0375	2.4837	0.0263	0.0651	2.5869	0.0769	0.1388	2.5544	0.0444	0.0742	2.6136	0.1036	0.1557
	*β*	0.8874	0.0674	0.0261	0.8573	0.0373	0.0354	0.8570	0.0370	0.0666	0.8574	0.0374	0.0429	0.8379	0.0179	0.0697
	*θ*	3.0133	0.0533	0.0252	2.9893	0.0293	0.0784	3.0435	0.0835	0.1455	3.0257	0.0657	0.0734	3.0228	0.0628	0.1287
	*λ*	0.0937	0.0263	0.0181	0.1139	0.0060	0.0446	0.1901	0.0701	0.0976	0.1536	0.0336	0.0436	0.2032	0.0832	0.1059
n = 500	*α*	2.4958	0.0142	0.0200	2.4877	0.0223	0.1191	2.5653	0.0553	0.0786	2.5422	0.0322	0.0398	2.5847	0.0747	0.0838
	*β*	0.8690	0.0490	0.0138	0.8481	0.0281	0.0373	0.8497	0.0297	0.0395	0.8538	0.0338	0.0284	0.8463	0.0263	0.0489
	*θ*	3.0054	0.0454	0.0168	2.9770	0.0170	0.0546	3.0344	0.0744	0.0913	3.0224	0.0624	0.0544	3.0313	0.0713	0.1118
	*λ*	0.1032	0.0168	0.0095	0.1072	0.0127	0.0272	0.1707	0.0507	0.0552	0.1442	0.0242	0.0226	0.1816	0.0616	0.0542

**Table 2:** Simulation study at α=2.85, β=1.47, θ=2.28, λ=0.065.

Set II:			MLE			PE			LSE			WLS			CVM	
		Est	Bias	MSE	Est	Bias	MSE	Est	Bias	MSE	Est	Bias	MSE	Est	Bias	MSE
n = 20	*α*	6.3942	3.5442	1774.0043	4.2659	1.4159	101.1328	11.2879	8.4379	21304.0329	7.2642	4.4142	2029.7813	8.4548	5.6048	2007.4598
	*β*	2.3283	0.8583	60.7686	1.7183	0.2488	10.0850	2.2286	0.7586	31.7398	1.9587	0.4887	25.5324	2.1788	0.7088	23.3568
	*θ*	3.8906	1.6106	376.2841	3.0611	0.7811	24.7363	6.7104	4.4304	5965.9924	4.4161	2.1361	492.2807	4.6709	2.3909	372.2855
	*λ*	1.5942	1.5292	374.4499	0.8503	0.7853	22.8928	4.5274	4.4624	5965.1149	2.3127	2.2477	492.4532	2.5363	2.4713	369.7120
n = 50	*α*	2.8914	0.0414	1.3736	3.0141	0.1641	3.4922	4.7897	1.9397	911.6313	3.3640	0.5140	5.3931	3.6741	0.8241	19.5162
	*β*	1.7760	0.3060	1.1214	1.5749	0.1049	0.6950	1.6449	0.1749	3.9078	1.5310	0.0610	2.6642	1.6645	0.1945	2.8779
	*θ*	2.3538	0.0738	0.2059	2.4068	0.1268	0.5657	3.1735	0.8935	186.2852	2.4699	0.1899	1.2402	2.6177	0.3377	4.9544
	*λ*	0.0408	0.0242	0.2397	0.1850	0.1200	0.6921	1.0183	0.9533	185.8847	0.3390	0.2740	1.3151	0.4548	0.3898	5.1099
n = 100	*α*	2.8113	0.0387	0.2392	2.8664	0.0186	0.3882	3.1741	0.3241	1.9889	3.0214	0.1714	0.6412	3.1438	0.2938	1.4829
	*β*	1.6685	0.1985	0.2822	1.5798	0.1097	0.4334	1.5591	0.0891	1.2777	1.5054	0.0354	0.3851	1.6067	0.1367	0.9251
	*θ*	2.3190	0.0390	0.0450	2.3584	0.0784	0.0838	2.4286	0.1486	0.5200	2.3498	0.0698	0.1242	2.4035	0.1235	0.3035
	*λ*	0.0232	0.0418	0.0474	0.1039	0.0389	0.0902	0.2465	0.1815	0.5121	0.1594	0.0944	0.1416	0.1967	0.1317	0.3337
n = 200	*α*	2.8223	0.0277	0.1050	2.8393	0.1068	0.1605	2.9963	0.1463	0.5075	2.9129	0.0629	0.2165	2.9891	0.1391	0.4392
	*β*	1.6194	0.1494	0.1224	1.5598	0.0898	0.1898	1.5630	0.0930	0.3906	1.5114	0.0414	0.1914	1.5533	0.0653	0.4472
	*θ*	2.3208	0.0408	0.0230	2.3305	0.0505	0.0466	2.3865	0.1065	0.1489	2.3241	0.0441	0.0641	2.3334	0.0543	0.1447
	*λ*	0.0381	0.0269	0.0204	0.0744	0.0094	0.0362	0.1518	0.0868	0.1233	0.1034	0.0384	0.0474	0.1292	0.0642	0.1019
n = 300	*α*	2.8256	0.0244	0.0664	2.8213	0.0287	0.1001	2.9423	0.0923	0.2871	2.8815	0.0315	0.1240	2.9525	0.1025	0.2554
	*β*	1.5995	0.1295	0.0806	1.5400	0.0701	0.1201	1.5310	0.0610	0.2410	1.5307	0.0607	0.1197	1.5339	0.0639	0.2806
	*θ*	2.3194	0.0394	0.0158	2.3133	0.0334	0.0323	2.3445	0.0645	0.0917	2.3237	0.0437	0.0383	2.3325	0.0525	0.0925
	*λ*	0.0436	0.0214	0.0125	0.0601	0.0049	0.0226	0.1204	0.0554	0.0693	0.0847	0.0197	0.0272	0.1128	0.0478	0.0589
n = 500	*α*	2.8319	0.0181	0.0379	2.8408	0.0092	0.0601	2.8924	0.0424	0.1396	2.8559	0.0059	0.0652	2.9316	0.0816	0.1431
	*β*	1.5649	0.0949	0.0412	1.5227	0.0527	0.0624	1.5279	0.0579	0.1374	1.5116	0.0416	0.0648	1.5339	0.0635	0.1388
	*θ*	2.3118	0.0318	0.0116	2.3094	0.0293	0.0196	2.3275	0.0475	0.0628	2.3045	0.0245	0.0275	2.3345	0.0545	0.0632
	*λ*	0.0511	0.0139	0.0070	0.0645	0.0004	0.0133	0.0940	0.0290	0.0338	0.0722	0.0072	0.0140	0.1025	0.0375	0.0330

**Table 3:** Simulation study at α=1.7, β=1.96, θ=1.5, λ=0.0035.

Set III:			MLE			PE			LSE			WLS			CVM	
		Est	Bias	MSE	Est	Bias	MSE	Est	Bias	MSE	Est	Bias	MSE	Est	Bias	MSE
n = 20	*α*	1.8676	0.1676	35.3950	1.8665	0.1665	3.3540	3.5367	1.8367	427.4955	2.4833	0.7833	76.7220	3.4396	1.7396	181.3917
	*β*	2.1491	0.1891	0.5407	1.9458	0.0142	0.4827	2.4873	0.5273	259.8537	1.8998	0.0602	1.1971	2.2222	0.2622	11.2306
	*θ*	1.6661	0.1661	3.0028	1.7050	0.2050	0.3172	2.1275	0.6275	43.6288	1.7547	0.2547	5.1495	1.9527	0.4527	17.1298
	*λ*	0.0313	0.0278	3.0175	0.0778	0.0743	0.2656	0.6341	0.6306	42.9895	0.2501	0.2466	5.2786	0.5145	0.5110	16.6786
n = 50	*α*	1.6319	0.0681	0.0838	1.6668	0.0331	0.1896	2.0140	0.3140	11.2312	1.9081	0.2081	15.1630	1.8767	0.1767	3.3547
	*β*	2.1248	0.1648	0.0988	2.0524	0.0924	0.1931	1.9998	0.0398	0.8968	2.0414	0.0814	4.2239	1.9729	0.0129	0.3057
	*θ*	1.5531	0.0531	0.0361	1.6049	0.1049	0.0535	1.6289	0.1289	1.3756	1.5839	0.0839	1.0238	1.5458	0.0458	0.0907
	*λ*	0.0284	0.0319	0.0048	0.0042	0.0007	0.0188	0.1182	0.1147	1.3562	0.0649	0.0614	0.9558	0.0530	0.0495	0.0302
n = 100	*α*	1.6508	0.0492	0.0311	1.6552	0.0448	0.0843	1.7877	0.0877	0.1374	1.7375	0.0375	0.0558	1.7839	0.0839	0.1120
	*β*	2.0977	0.1377	0.0607	2.0500	0.0900	0.0911	1.9970	0.0370	0.2049	2.0124	0.0524	0.0754	2.0084	0.0484	0.1268
	*θ*	1.5474	0.0474	0.0172	1.5682	0.0683	0.0225	1.5503	0.0503	0.0321	1.5428	0.0428	0.0196	1.5375	0.0375	0.0282
	*λ*	0.0164	0.0199	0.0016	0.0023	0.0058	0.0084	0.0378	0.0343	0.0125	0.0172	0.0137	0.0037	0.0274	0.0239	0.0097
n = 200	*α*	1.6661	0.0339	0.0149	1.6603	0.0397	0.0409	1.7468	0.0468	0.0455	1.7180	0.0180	0.0223	1.7585	0.0585	0.0461
	*β*	2.0609	0.1009	0.0258	2.0429	0.0829	0.0418	2.0097	0.0497	0.0692	2.0207	0.0607	0.0367	2.0171	0.0571	0.0715
	*θ*	1.5350	0.0350	0.0073	1.5453	0.0454	0.0105	1.5352	0.0352	0.0137	1.5321	0.0321	0.0085	1.5336	0.0336	0.0142
	*λ*	0.0088	0.0123	0.0007	0.0057	0.0092	0.0041	0.0215	0.0180	0.0038	0.0098	0.0063	0.0013	0.0207	0.0172	0.0036
n = 300	*α*	1.6749	0.0251	0.0090	1.6599	0.0401	0.0287	1.7359	0.0359	0.0261	1.7130	0.0130	0.0132	1.7415	0.0415	0.0250
	*β*	2.0445	0.0845	0.0179	2.0411	0.0811	0.0353	2.0153	0.0553	0.0436	2.0103	0.0503	0.0202	2.0106	0.0506	0.0378
	*θ*	1.5313	0.0313	0.0049	1.5441	0.0441	0.0088	1.5339	0.0339	0.0092	1.5256	0.0256	0.0052	1.5287	0.0287	0.0085
	*λ*	0.0052	0.0087	0.0003	0.0074	0.0109	0.0029	0.0171	0.0136	0.0021	0.0079	0.0044	0.0007	0.0164	0.0129	0.0019
n = 500	*α*	1.6800	0.0200	0.0052	1.6786	0.0214	0.0168	1.7242	0.0242	0.0134	1.7067	0.0067	0.0072	1.7329	0.0329	0.0158
	*β*	2.0928	0.0698	0.0112	2.0187	0.0587	0.0168	2.0146	0.0546	0.0257	2.0093	0.0493	0.0162	2.0130	0.0539	0.0329
	*θ*	1.5273	0.0273	0.0029	1.5305	0.0305	0.0046	1.5285	0.0285	0.0049	1.5229	0.0229	0.0033	1.5281	0.0281	0.0062
	*λ*	0.0026	0.0061	0.0002	0.0028	0.0063	0.0017	0.0130	0.0095	0.0010	0.0062	0.0027	0.0003	0.0148	0.0113	0.0012

**Table 4:** Simulation study at α=2.2, β=1.6, θ=1.95, λ=0.0075.

Set IV:			MLE			PE			LSE			WLS			CVM	
		Est	Bias	MSE	Est	Bias	MSE	Est	Bias	MSE	Est	Bias	MSE	Est	Bias	MSE
n = 20	*α*	2.5315	0.3315	32.5347	3.1397	0.9397	190.8599	4.5398	2.3398	478.7237	4.5916	2.3916	539.7621	4.1114	1.9114	355.4171
	*β*	1.9033	0.3033	0.7656	2.0855	0.4855	150.6772	2.0545	0.4545	45.0686	1.8984	0.2984	30.8118	1.9426	0.3426	10.0502
	*θ*	2.1620	0.2120	5.0321	2.3988	0.4488	22.6806	3.1193	1.1693	145.4113	2.9626	1.0126	123.9859	2.6731	0.7231	71.2345
	*λ*	0.0740	0.0665	5.1789	0.4117	0.4041	22.2126	1.1840	1.1765	144.2511	1.1038	1.0963	125.4434	0.7855	0.7780	70.6868
n = 50	*α*	2.1840	0.0160	0.3674	2.2807	0.0807	2.5614	2.8778	0.6778	67.1403	2.4236	0.2236	1.0014	2.6019	0.4019	2.3763
	*β*	1.7853	0.1853	0.2241	1.7172	0.1172	4.3738	1.6333	0.0333	1.1406	1.5944	0.0056	0.3486	1.6808	0.0808	1.5287
	*θ*	2.0032	0.0532	0.0537	2.0762	0.1262	0.4852	2.2224	0.2724	9.9731	2.0350	0.0850	0.1487	2.0636	0.1136	0.4249
	*λ*	0.0309	0.0384	0.0477	0.0611	0.0536	0.4358	0.3103	0.3028	9.9790	0.1104	0.1029	0.1663	0.1718	0.1643	0.4516
n = 100	*α*	2.1672	0.0328	0.0896	2.1880	0.0119	0.1757	2.3653	0.1653	0.4232	2.2924	0.0924	0.1951	2.3634	0.1634	0.3799
	*β*	1.7411	0.1411	0.0946	1.6830	0.0830	0.1821	1.6355	0.0355	0.3536	1.6226	0.0226	0.1792	1.6302	0.0302	0.2941
	*θ*	1.9972	0.0472	0.0187	2.0175	0.0675	0.0457	2.0249	0.0749	0.1103	1.9983	0.0483	0.0475	1.9981	0.0481	0.0861
	*λ*	0.0211	0.0286	0.0109	0.0118	0.0043	0.0322	0.0901	0.0826	0.0829	0.0487	0.0412	0.0318	0.0729	0.0654	0.0702
n = 200	*α*	2.1772	0.0228	0.0377	2.1609	0.0390	0.0761	2.2897	0.0897	0.1553	2.2516	0.0516	0.0762	2.2825	0.0825	0.1298
	*β*	1.7121	0.1121	0.0464	1.6983	0.0985	0.0974	1.6232	0.0232	0.1787	1.6500	0.0500	0.0763	1.6625	0.0625	0.1533
	*θ*	1.9902	0.0402	0.0092	2.0103	0.0603	0.0204	1.9904	0.0404	0.0447	1.9984	0.0484	0.0226	1.9969	0.0469	0.0432
	*λ*	0.0096	0.0171	0.0044	0.0010	0.0085	0.0140	0.0508	0.0433	0.0277	0.0302	0.0227	0.0118	0.0424	0.0349	0.0239
n = 300	*α*	2.1801	0.0199	0.0227	2.1835	0.0165	0.0488	2.2587	0.0587	0.0885	2.2325	0.0325	0.0427	2.2596	0.0596	0.0757
	*β*	1.6926	0.0926	0.0296	1.6591	0.0591	0.0549	1.6527	0.0527	0.0961	1.6445	0.0445	0.0497	1.6703	0.0703	0.1161
	*θ*	1.9846	0.0346	0.0061	1.9880	0.0380	0.0163	1.9992	0.0492	0.0344	1.9864	0.0364	0.0175	1.9963	0.0463	0.0323
	*λ*	0.0061	0.0136	0.0026	0.0055	0.0020	0.0080	0.0373	0.0298	0.0157	0.0227	0.0152	0.0062	0.0321	0.0246	0.0138
n = 500	*α*	2.1839	0.0161	0.0138	2.1689	0.0310	0.0287	2.2479	0.0479	0.0476	2.2458	0.0458	0.0482	2.2466	0.0466	0.0443
	*β*	1.6732	0.0732	0.0198	1.6694	0.0694	0.0398	1.6521	0.0521	0.0529	1.6397	0.0397	0.0559	1.6643	0.0643	0.0482
	*θ*	1.9777	0.0277	0.0049	1.9845	0.0345	0.0115	1.9945	0.0445	0.0219	1.9886	0.0386	0.0226	1.9935	0.0435	0.0210
	*λ*	0.0021	0.0096	0.0015	0.0016	0.0091	0.0053	0.0313	0.0238	0.0085	0.0307	0.0232	0.0088	0.0275	0.0200	0.0080

**Table 5:** Simulation study at α=3.7, β=3.87, θ=3.9, λ=0.22.

Set V:			MLE			PE			LSE			WLS			CVM	
		Est	Bias	MSE	Est	Bias	MSE	Est	Bias	MSE	Est	Bias	MSE	Est	Bias	MSE
n = 20	*α*	11.6219	7.9219	6170.698	9.8465	6.1465	1887.8681	11.3522	7.6522	6687.8553	12.4827	8.7827	4099.6328	14.1620	10.4620	3002.625
	*β*	9.4330	5.5630	3127.177	8.0629	4.1929	1671.7648	5.8798	2.0098	198.7719	9.3433	5.4733	2382.6693	7.7782	3.9082	487.4371
	*θ*	8.0571	4.1571	1553.689	7.6275	3.7275	590.9027	8.1237	4.2237	1517.3507	8.6366	4.7366	801.0214	8.7809	4.8809	610.2051
	*λ*	4.0088	3.7888	1497.414	3.8169	3.5969	578.3639	4.3431	4.1231	1500.5377	4.7494	4.5294	746.7298	5.1470	4.9270	607.0535
n = 50	*α*	5.2467	1.5467	554.8111	5.0942	1.3942	231.9802	6.3443	2.6443	189.5234	5.3637	1.6637	135.4818	5.9091	2.2091	161.4681
	*β*	4.5285	0.6585	119.8566	4.5338	0.6638	112.4696	5.1926	1.3226	137.4825	4.5988	0.7288	61.9959	5.3374	1.4674	139.9609
	*θ*	4.6562	0.7562	151.2397	4.6587	0.7587	50.1429	5.3805	1.4805	65.1351	4.8170	0.9170	49.3651	5.0220	1.1220	53.9379
	*λ*	0.9614	0.7414	154.1699	1.0075	0.7875	49.8083	1.7795	1.5595	64.2530	1.2047	0.9847	48.7744	1.4361	1.2161	52.2652
n = 100	*α*	3.7845	0.0845	2.1446	3.8719	0.1719	1.2455	4.9342	1.2342	117.5396	4.1113	0.4113	3.3061	4.4775	0.7775	13.7876
	*β*	4.1792	0.3092	2.8558	3.9452	0.0752	2.8975	4.4491	0.5791	23.7011	4.0461	0.1761	4.4843	4.2742	0.4042	17.2376
	*θ*	3.9658	0.0658	0.4326	4.0492	0.1492	0.3258	4.6301	0.7301	43.2867	4.1186	0.2186	0.8375	4.2698	0.3698	4.1075
	*λ*	0.2340	0.0140	0.5626	0.3603	0.1403	0.3997	0.9764	0.7564	43.2612	0.4681	0.2481	0.9661	0.6577	0.4377	4.3296
n = 200	*α*	3.6801	0.0199	0.3381	3.7914	0.0913	0.4653	4.0379	0.3379	1.9430	3.8448	0.1448	0.6958	4.0244	0.3244	1.7605
	*β*	4.1037	0.2337	0.8252	3.9063	0.0362	1.1489	4.1968	0.3268	4.5192	4.0910	0.2210	2.2477	4.2236	0.3536	5.7488
	*θ*	3.9354	0.0354	0.0838	3.9777	0.0777	0.1260	4.1159	0.2159	0.6075	4.0140	0.1140	0.2281	4.0727	0.1727	0.5812
	*λ*	0.1944	0.0256	0.1005	0.2896	0.0696	0.1413	0.4267	0.2067	0.6260	0.3082	0.0882	0.2142	0.4014	0.1814	0.5561
n = 300	*α*	3.6853	0.0147	0.2172	3.7176	0.0177	0.2459	3.9207	0.2207	1.0848	3.7849	0.0849	0.3988	3.9173	0.2173	1.0670
	*β*	4.0788	0.2088	0.5494	4.0007	0.1307	0.7560	4.1122	0.2422	2.7954	4.0568	0.1868	1.3895	4.2230	0.3530	4.9828
	*θ*	3.9345	0.0345	0.0560	3.9692	0.0692	0.1009	4.0464	0.1464	0.3834	3.9806	0.0806	0.1383	4.0417	0.1417	0.3729
	*λ*	0.2008	0.0192	0.0618	0.2465	0.0265	0.0742	0.3577	0.1377	0.3510	0.2723	0.0532	0.1216	0.3440	0.1240	0.3359
n = 500	*α*	3.6962	0.0038	0.1224	3.7091	0.0091	0.1477	3.8260	0.1260	0.5018	3.7455	0.0455	0.2144	3.8207	0.1207	0.4761
	*β*	4.0176	0.1476	0.2981	3.9938	0.1237	0.3869	4.1591	0.2891	2.0788	4.0069	0.1369	0.8403	4.1374	0.2674	2.6406
	*θ*	3.9293	0.0293	0.0362	3.9582	0.0582	0.0588	4.0173	0.1173	0.2235	3.9511	0.0511	0.0951	3.9918	0.0918	0.2200
	*λ*	0.2110	0.0090	0.0340	0.2388	0.0188	0.0455	0.2986	0.0786	0.1604	0.2486	0.0286	0.0647	0.2889	0.0689	0.1505

**Fig 4:**
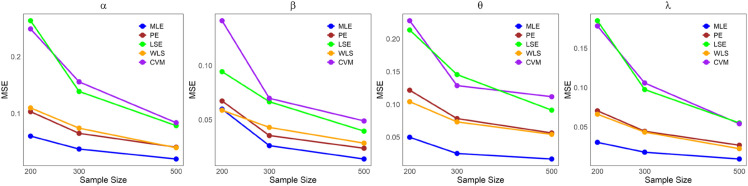
Comparing the MSEs of the estimation methods by sample size for Table 1.

**Fig 5:**
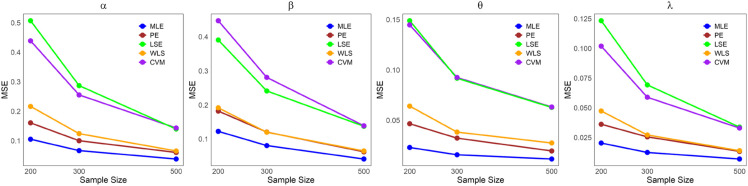
Comparing the MSEs of the estimation methods by sample size for Table 2.

**Fig 6:**
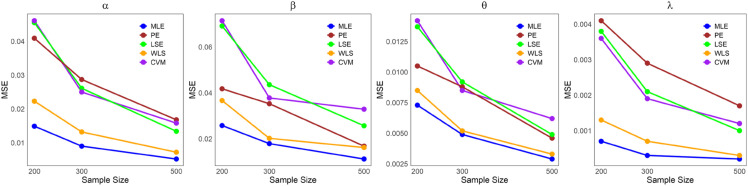
Comparing the MSEs of the estimation methods by sample size for Table 3.

**Fig 7:**
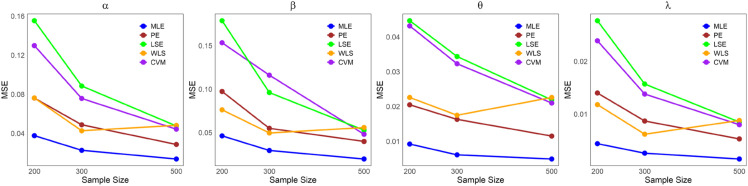
Comparing the MSEs of the estimation methods by sample size for Table 4.

**Fig 8:**
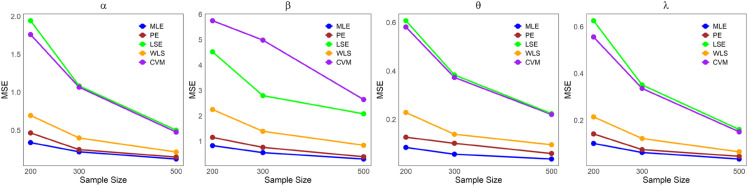
Comparing the MSEs of the estimation methods by sample size for Table 5.

The values in Tables 1–5 demonstrate that the average estimates of the LKME parameters approach their actual values as the sample size increases. This finding indicates that the estimation becomes more accurate as the dataset size increases. Furthermore, Figs 4–8 show comparative results of the different estimation methods, demonstrating their mean squared errors (MSE) under large sample sizes (n=200, 300, and 500). As indicated by these figures, compared with all the other estimation methods, the ML method exhibits superior performance and provides the most accurate estimates. This is succeeded by the PE and WLE methods for most circumstances. Moreover, except for parameters *α* and *λ* in case 3, it is clear that the LSE and CVM are the least precise methods among all the examined methods, as they yield the highest MSEs. All the statistical analyses were performed in R Statistical Software version 4.3.2 (R Core Team, 2023) [21].

## 6 Applications

The LKME model was used to fit real-world datasets, and it outperformed the comparison distributions on the five datasets. Here is a brief overview of the dataset.

**Service Time Dataset**

The first dataset is extracted from [22], and contains 63 recorded service times for the aircraft windshields.

0.046, 1.436, 2.592, 0.140, 1.492, 2.600, 0.150, 1.580, 2.670, 0.248, 1.719, 2.717, 0.280, 1.794, 2.819, 0.313, 1.915, 2.820, 0.389, 1.920, 2.878, 0.487, 1.963, 2.950, 0.622, 1.978, 3.003, 0.900, 2.053, 3.102, 0.952, 2.065, 3.304, 0.996, 2.117, 3.483, 1.003, 2.137, 3.500, 1.010, 2.141, 3.622, 1.085, 2.163, 3.665, 1.092, 2.183, 3.695, 1.152, 2.240, 4.015, 1.183, 2.341, 4.628, 1.244, 2.435, 4.806, 1.249, 2.464, 4.881, 1.262, 2.543, 5.140.

**Breaking stress of carbon fibers.**

The second dataset includes 100 observations and was derived from [23],

3.70,2.74, 2.73, 2.50, 3.60, 3.11, 3.27, 2.87, 1.47, 3.11, 4.42, 2.41, 3.19,3.22, 1.69, 3.28, 3.09, 1.87, 3.15, 4.90, 3.75, 2.43, 2.95, 2.97, 3.39, 2.96, 2.53, 2.67, 2.93, 3.22, 3.39,2.81, 4.20, 3.33, 2.55, 3.31, 3.31, 2.85, 2.56, 3.56, 3.15, 2.35, 2.55, 2.59, 2.38, 2.81, 2.77, 2.17, 2.83,1.92, 1.41, 3.68, 2.97, 1.36, 0.98, 2.76, 4.91, 3.68, 1.84, 1.59, 3.19, 1.57, 0.81, 5.56, 1.73, 1.59, 2.00,1.22, 1.12, 1.71, 2.17, 1.17, 5.08, 2.48, 1.18, 3.51, 2.17, 1.69, 1.25, 4.38, 1.84, 0.39, 3.68, 2.48, 0.85, 1.61, 2.79, 4.70, 2.03, 1.80, 1.57, 1.08, 2.03, 1.61, 2.12, 1.89, 2.88, 2.82, 2.05, 3.65.

**The tensile strength.**

The third dataset, containing 69 observations, is obtained from [24] and is given by:

0.312, 0.314, 0.479, 0.552, 0.700, 0.803, 0.861, 0.865, 0.944, 0.958, 0.966, 0.997, 1.006, 1.021, 1.027, 1.055, 1.063, 1.098, 1.140, 1.179, 1.224, 1.240, 1.253, 1.270, 1.272, 1.274, 1.301, 1.301, 1.359, 1.382, 1.382, 1.426, 1.434, 1.435, 1.478, 1.490, 1.511, 1.514, 1.535, 1.554, 1.566, 1.570, 1.586, 1.629, 1.633, 1.642, 1.648, 1.684, 1.697, 1.726, 1.770, 1.773, 1.800, 1.809, 1.818, 1.821, 1.848, 1.880, 1.954, 2.012, 2.067, 2.084, 2.090, 2.096, 2.128, 2.233, 2.433, 2.585, 2.585.

**Single fibers with gauge lengths of 20mm.**

The fourth dataset is obtained from [13] and contains 69 observations:

1.312, 1.314, 1.479, 1.552, 1.7, 1.803, 1.861, 1.865, 1.944, 1.958, 1.966, 1.997, 2.006, 2.021, 2.027, 2.055, 2.063, 2.098, 2.14, 2.179, 2.224, 2.24, 2.253, 2.27, 2.272, 2.274, 2.301, 2.301, 2.359, 2.382, 2.382, 2.426, 2.434, 2.435, 2.478, 2.49, 2.511, 2.514, 2.535, 2.554, 2.566, 2.57, 2.586, 2.629, 2.633, 2.642, 2.648, 2.684, 2.697, 2.726, 2.77, 2.773, 2.8, 2.809, 2.818, 2.821, 2.848, 2.88, 2.954, 3.012, 3.067, 3.084, 3.09, 3.096, 3.128, 3.233, 3.433, 3.585, 3.858.

**Single fibers with gauge lengths of 50mm.**

The fifth dataset was sourced from [13], and contains 69 observations:

0.562, 0.564, 0.729, 0.802, 0.95, 1.053, 1.111, 1.115, 1.194, 1.208, 1.216, 1.247, 1.256, 1.271, 1.277, 1.305, 1.313, 1.348, 1.39, 1.429, 1.474, 1.49, 1.503, 1.52, 1.522, 1.524, 1.551, 1.551, 1.609, 1.632, 1.632, 1.676, 1.684, 1.685, 1.728, 1.74, 1.761, 1.764, 1.785, 1.804, 1.816, 1.824, 1.836, 1.879, 1.883, 1.892, 1.898, 1.934, 1.947, 1.976, 2.02, 2.023, 2.05, 2.059, 2.068, 2.071, 2.098, 2.13, 2.204, 2.317, 2.334, 2.34, 2.346, 2.378, 2.483, 2.683, 2.835, 2.835, 2.262.

The fitting performance of the LKME model across the five datasets is assessed by comparison with the fits of the following distributions.

- Lomax distribution with the CDF function:
  $$G(x)=1-{\left[1+\frac{x}{\lambda }\right]}^{-\theta },\;\theta ,\lambda >0.$$
- The Kavya-Manoharan exponential (KME) distribution with the CDF function is given in (3).
- The Kavya–Manoharan generalized exponential distribution (KMGE) by [14]
  $$F(x;\theta ,\lambda )=\frac{e}{e-1}\left[1-e^{-{\left(1-e^{-\lambda x}\right)}^{\theta }}\right],\;x>0,\;\theta ,\lambda >0.$$
- The Kavya–Manoharan transformation inverse length biased exponential (KMILBE) by [15]
  $$F(x;\theta )=\frac{e}{e-1}\left[1-e^{-\left(1+\frac{\theta }{x}\right)e^{-\frac{\theta }{x}}}\right],\;x>0,\;\theta >0.$$
- The alpha power transformed generalized Lomax (APTGL) distribution, by [25]
  $$F(x)=\left\{ \begin{array}{ccc} \frac{\alpha^{{\left(1-{\left(1+\frac{x}{\lambda }\right)}^{-\theta }\right)}^{\beta }}-1}{\alpha -1} & if & \alpha \neq 1\\ {\left(1-{\left(1+\frac{x}{\lambda }\right)}^{-\theta }\right)}^{\beta } & if & \alpha =1 \end{array}\right.\;,$$
  where α,β,θ,λ>0.

We computed several goodness-of-fit (GoF) metrics, namely, the Akaike information criterion (AIC), Bayesian information criterion (BIC), consistent Akaike information criterion (CAIC), Hannan Quinn information criterion (HQIC), Anderson Darling (A-D) test and Kolmogorov Smirnov (KS) statistic, with their corresponding p values to compare these distributions with those of the LKME model.

Tables 6–10 demonstrate that the fit of the LKME model is superior to that of the other distributions for the five datasets. Compared with the other fitted models, the LKME model has the lowest GoF metric values and the highest p value. This finding verifies that the LKME model outperforms rival distributions and demonstrates its efficacy in appropriately describing the given datasets.

**Table 6:** GOF criteria for service time.

Model	*L*	AIC	BIC	CAIC	HQIC	K-S	p-value	A-D
LKME	100.1518	208.3036	216.8761	208.9932	211.6752	0.0859	0.7081	0.3534
Lomax	109.2986	222.5972	226.8835	222.7972	224.2830	0.2076	0.0074	3.8755
KME	113.0619	228.1238	230.2670	228.1894	228.9667	0.2227	0.0032	4.5464
KMGE	105.2093	214.4186	218.7049	214.6186	216.1044	0.1467	0.1201	1.5166
KMILBE	168.7221	339.4442	341.5873	339.5097	340.2871	0.4323	3.242$\times 10^{-11}$	26.9640
APTGL	100.5127	209.0253	217.5979	209.7150	212.3970	0.0949	0.5884	0.5522

**Table 7:** GOF criteria for the breaking stress of carbon fibres.

Model	*L*	AIC	BIC	CAIC	HQIC	K-S	p-value	A-D
LKME	141.4022	290.8045	301.2252	291.2255	295.0219	0.0638	0.8102	0.4246
Lomax	196.3709	396.7417	401.9521	396.8654	398.8504	0.3205	2.385$\times 10^{-9}$	17.2970
KME	204.6995	411.3899	414.0041	411.4397	412.4533	0.3190	2.888$\times 10^{-9}$	17.7930
KMGE	147.5455	299.0911	304.3014	299.2148	301.1998	0.1095	0.1819	1.3797
KMILBE	168.7664	339.5327	342.1379	339.5735	340.5871	0.2142	0.0002	5.6964
APTGL	142.8936	293.7871	304.2078	294.2082	298.0045	0.0807	0.5326	0.6509

**Table 8:** GOF criteria for the tensile strength data.

Model	*L*	AIC	BIC	CAIC	HQIC	K-S	p-value	A-D
LKME	48.8142	105.6284	114.5648	106.2534	109.1738	0.0433	0.9995	0.1640
Lomax	94.7013	193.4026	197.8708	193.5844	195.1753	0.3624	2.693$\times 10^{-8}$	13.7090
KME	100.6840	203.3679	205.6020	203.4276	204.2543	0.3576	4.347$\times 10^{-8}$	13.8370
KMGE	57.7346	119.4692	123.9374	119.6510	121.2419	0.1071	0.4068	1.5832
KMILBE	75.2421	152.4843	154.7184	152.5440	153.3706	0.2372	0.0009	5.6119
APTGL	52.0720	112.1440	121.0804	112.7690	115.6894	0.0658	0.9266	0.5816

**Table 9:** GOF criteria for the single fibers with gauge lengths of 20mm.

Model	*L*	AIC	BIC	CAIC	HQIC	K-S	p-value	A-D
LKME	50.3967	108.7933	117.7297	109.4183	112.3387	0.0504	0.9947	0.2153
Lomax	130.9789	265.9578	270.4260	266.1396	267.7305	0.4477	1.952$\times 10^{-12}$	20.3110
KME	137.5526	277.1052	279.3393	277.1649	277.9915	0.4343	9.989$\times 10^{-12}$	19.8030
KMGE	55.7874	115.5747	120.0429	115.7565	117.3474	0.0911	0.6153	1.1062
KMILBE	99.2810	200.5621	202.7962	200.6218	201.4484	0.3767	6.282$\times 10^{-9}$	13.0780
APTGL	51.9231	111.8461	120.7825	112.4711	115.3915	0.0603	0.9634	0.4347

**Table 10:** GOF criteria for the single fibers with gauge lengths of 50mm.

Model	*L*	AIC	BIC	CAIC	HQIC	K-S	p-value	A-D
LKME	48.8160	105.6320	114.5684	106.2570	109.1774	0.0434	0.9995	0.1637
Lomax	105.6698	215.3396	219.8078	215.5214	217.1123	0.3926	1.159$\times 10^{-9}$	16.0300
KME	111.9074	225.8149	228.0490	225.8746	226.7012	0.3852	2.570$\times 10^{-9}$	15.9540
KMGE	56.4053	116.8105	121.2787	116.9924	118.5832	0.0984	0.5161	1.3449
KMILBE	79.3036	160.6071	162.8412	160.6668	161.4935	0.2925	1.491$\times 10^{-5}$	7.7659
APTGL	51.3630	110.7259	119.6623	111.3509	114.2713	0.0619	0.9542	0.4884

**Fig 9:**
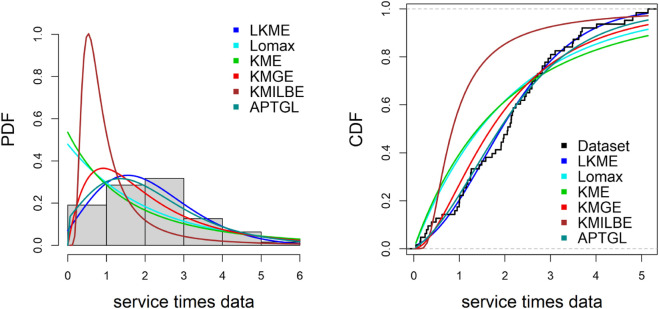
Estimated PDF and CDF for the service time data.

**Fig 10:**
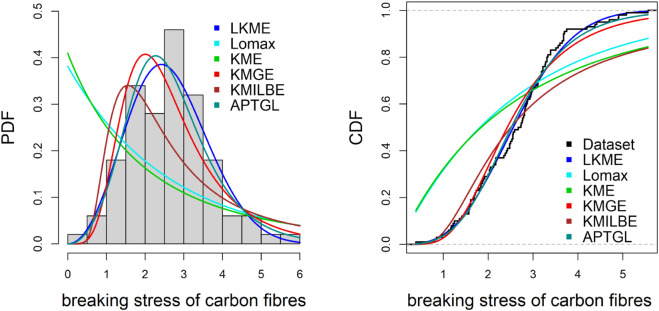
Estimated PDF and CDF for the breaking stress of carbon fibers.

**Fig 11:**
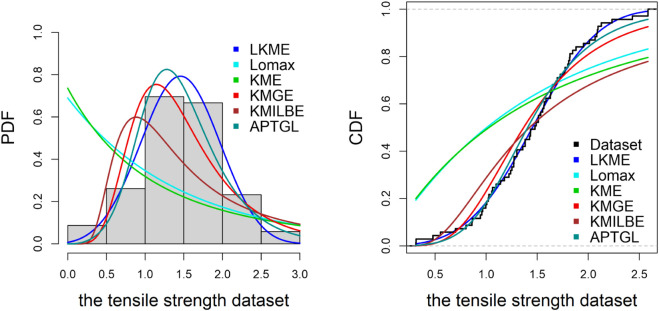
Estimated PDF and CDF for the tensile strength data.

**Fig 12:**
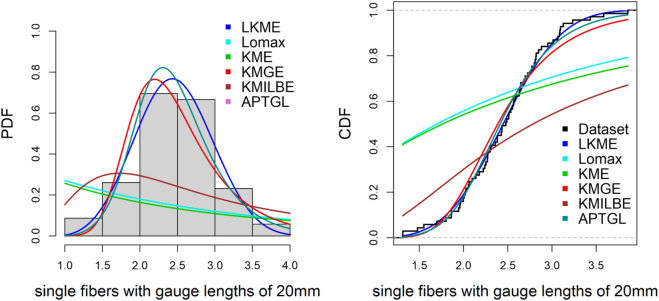
Estimated PDF and CDF for single fibers with gauge lengths of 20 mm.

**Fig 13:**
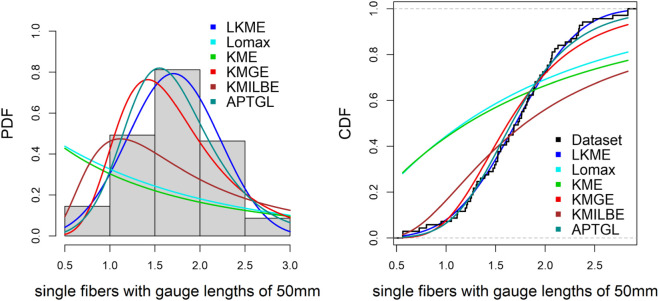
Estimated PDF and CDF for single fibers with gauge lengths of 50 mm.

Furthermore, Figs 9–13 show the fitted PDF and CDF for each dataset. These plots indicate that compared with the other models, the LKME model is the most effective at capturing the data skewness.

## 7 Conclusions

In this paper, we present a new member of the Lomax-G family called the Lomax Kavya Manoharan exponential distribution. The LKME density can be left-skewed, right-skewed, symmetric, semi-symmetric, or inverted J-shaped. In addition, the hazard function can exhibit a broad spectrum of asymmetric patterns, including increasing, decreasing, J shape, and inverted J shape with different tail behaviors, allowing applications to real-world data. The statistical properties of the LKME distribution, such as quantiles, medians, moments, characteristic functions, order statistics, and R’enyi and Shannon entropies, were calculated. The LKME model’s parameters were estimated using five distinct estimation techniques: MLE, PE, LSE, WLS, and CVM. Monte Carlo simulations were performed to assess the accuracy and reliability of the parameter estimations, which revealed that the ML approach outperforms all the other estimation methods, yielding the most precise values. The superior fit of the LKME model over five real-world datasets compared with that of competing distributions demonstrates its applicability to real-world scenarios. These results highlight the remarkable ability of the LKME distribution to model and understand complex phenomena across multiple domains.

## Supporting information

S1 Appendix Order statistics. (PDF)

S2 Appendix Renyi entropy. (PDF)

S3 Appendix Shannon entropy. (PDF)
