# Supplementary material for: Analysis of engineering data with an innovative generalization of the Lomax distribution
Source: PLoS One. 2025 Oct 27;20(10):e0334323. doi: 10.1371/journal.pone.0334323 (PMC12558503; doi:10.1371/journal.pone.0334323)
Supplement: S1 Appendix — (PDF) [file pone.0334323.s001.pdf]

Appendix A: Order Statistics

The detailed proof of the LKME's order statistics given in Eq. (33) is as follows:

$$f_{r:n}(x) = \frac{f(x)}{\beta(r, n-r+1)} \sum_{v=0}^{n-r} (-1)^v \binom{n-r}{v} F^{v+r-1}(x).$$

By substituting Eqs. (5) and (11), we obtain

$$f_{r:n}(x) = \frac{\alpha\beta}{\beta(r, n-r+1)} \sum_{i=0}^{\infty} \sum_{j=0}^{\alpha-1} \sum_{v=0}^{n-r} (-1)^v \binom{n-r}{v} \binom{\alpha-1}{j} \frac{e^{v+r-1}}{(e-1)^{v+r}} \frac{\lambda^{\alpha-j-1}}{i!\,\theta^\alpha}$$
$$x^j e^{-\beta(i+1)\left(\frac{x+\lambda}{\theta}\right)^\alpha} \left[1 - e^{-\left(1-e^{-\beta\left(\frac{x+\lambda}{\theta}\right)^\alpha}\right)}\right]^{v+r-1}. \quad (1)$$

Expanding $\left[1 - e^{-\left(1-e^{-\beta\left(\frac{x+\lambda}{\theta}\right)^\alpha}\right)}\right]^{v+r-1}$ using the series expansion (18), we obtain

$$\left[1 - e^{-\left(1-e^{-\beta\left(\frac{x+\lambda}{\theta}\right)^\alpha}\right)}\right]^{v+r-1} = \sum_{k=0}^{v+r-1} (-1)^k \binom{v+r-1}{k} e^{-k\left(1-e^{-\beta\left(\frac{x+\lambda}{\theta}\right)^\alpha}\right)}.$$

After the exponential series expansion (28) is applied to the term $e^{-k\left(1-e^{-\beta\left(\frac{x+\lambda}{\theta}\right)^\alpha}\right)}$, we obtain

$$\left[1 - e^{-\left(1-e^{-\beta\left(\frac{x+\lambda}{\theta}\right)^\alpha}\right)}\right]^{v+r-1} = \sum_{k=0}^{v+r-1} \sum_{l=0}^{\infty} \frac{(-1)^{k+l}}{l!} k^l \binom{v+r-1}{k} \left[1 - e^{-\beta\left(\frac{x+\lambda}{\theta}\right)^\alpha}\right]^l.$$

Applying expansion (18) to the term $\left[1 - e^{-\beta\left(\frac{x+\lambda}{\theta}\right)^\alpha}\right]^l$, we obtain

$$\left[1 - e^{-\left(1-e^{-\beta\left(\frac{x+\lambda}{\theta}\right)^\alpha}\right)}\right]^{v+r-1} = \sum_{k=0}^{v+r-1} \sum_{l=0}^{\infty} \sum_{m=0}^{l} \frac{(-1)^{k+l+m}}{l!} k^l \binom{l}{m} \binom{v+r-1}{k} e^{-m\beta\left(\frac{x+\lambda}{\theta}\right)^\alpha}.$$

Substituting in (51), we obtain

$$f_{r:n}(x) = \frac{\alpha\beta}{\beta\left(r, n-r+1\right)} \sum_{i=0}^{\infty} \sum_{j=0}^{\alpha-1} \sum_{v=0}^{n-r} \sum_{k=0}^{v+r-1} \sum_{l=0}^{\infty} \sum_{m=0}^{l} \frac{(-1)^{k+l+m+v}}{i!\,l!} \binom{n-r}{v} \binom{\alpha-1}{j}$$
$$\binom{l}{m} \binom{v+r-1}{k} \frac{\lambda^{\alpha-j-1} k^l}{\theta^\alpha} \frac{e^{v+r-1}}{(e-1)^{v+r}} x^j e^{-\beta(m+i+1)\left(\frac{x+\lambda}{\theta}\right)^\alpha}.$$
