# Supplementary material for: Analysis of engineering data with an innovative generalization of the Lomax distribution
Source: PLoS One. 2025 Oct 27;20(10):e0334323. doi: 10.1371/journal.pone.0334323 (PMC12558503; doi:10.1371/journal.pone.0334323)
Supplement: S2 Appendix — (PDF) [file pone.0334323.s002.pdf]

Appendix B: Renyi Entropy

follows:

$$RE_x(u) = \frac{1}{1-u} log \left(\int_{-\infty}^{\infty} f^u(x)dx \right), \qquad u > 0, u \neq 1.$$

By substituting Eq. (6), we obtain.

$$\int_{-\lambda}^{\infty} f^u(x)dx = \left[\frac{\alpha\beta}{\theta(e-1)} \right]^u \int_{-\lambda}^{\infty} \left(\frac{x+\lambda}{\theta} \right)^{u(\alpha-1)} e^{-u\beta(\frac{x+\lambda}{\theta})^\alpha} e^{ue^{-\beta(\frac{x+\lambda}{\theta})^\alpha}} dx.$$

Using the expansion of the exponential series in Eq. (9), we obtain

$$\int_{-\lambda}^{\infty} f^u(x)dx = \left[\frac{\alpha\beta}{\theta(e-1)} \right]^u \sum_{i=0}^{\infty} \frac{u^i}{i!} \int_{-\lambda}^{\infty} \left(\frac{x+\lambda}{\theta} \right)^{u(\alpha-1)} e^{-\beta(i+u)(\frac{x+\lambda}{\theta})^\alpha} dx.$$

Substituting $w = \beta(i+u)\left(\frac{x+\lambda}{\theta}\right)^\alpha$, we obtain

$$\int_{-\lambda}^{\infty} f^u(x)dx = \left[\frac{\alpha\beta}{\theta\,(e-1)} \right]^u \sum_{i=0}^{\infty} \frac{u^i}{i!} \frac{\theta}{\alpha\beta(i+u)} \int_{0}^{\infty} \left[\frac{w}{\beta(i+u)} \right]^{\frac{u\alpha-u+1}{\alpha}-1} e^{-w} dw.$$

To simplify, we replace $\frac{u\alpha-u+1}{\alpha} = k$

$$\int_{-\lambda}^{\infty} f^u(x)dx = \left(\frac{\alpha}{\theta} \right)^{u-1} \frac{\beta^{u-k}}{(e-1)^u} \sum_{i=0}^{\infty} \frac{u^i}{i!} \frac{\Gamma(k)}{(i+u)^k}.$$

Therefore,

$$RE_x(u) = log\left(\frac{\alpha}{\theta}\right) + \frac{1}{u-1}\left[(u-k)\, log\beta - ulog\,(e-1) + \sum_{i=0}^{\infty} log\left(\frac{u^i}{i!\,(i+u)^k} \right) + \log\left(\Gamma(k)\right) \right]$$
