# Supplementary material for: Analysis of engineering data with an innovative generalization of the Lomax distribution
Source: PLoS One. 2025 Oct 27;20(10):e0334323. doi: 10.1371/journal.pone.0334323 (PMC12558503; doi:10.1371/journal.pone.0334323)
Supplement: S3 Appendix — (PDF) [file pone.0334323.s003.pdf]

Appendix C: Shannon Entropy

The detailed proof of the Shannon entropy of the LKME distribution given in Eq. (36) is as follows:

$$SE_x = -E\left[log\left(f(x)\right)\right] = -\int_{-\lambda}^{\infty} logf(x)\left[f(x)\right]dx.$$

By substituting Eq. (6), we obtain

$$SE_x = -\int_{-\lambda}^{\infty}\left[\frac{\alpha\beta}{\theta(e-1)}\left(\frac{x+\lambda}{\theta}\right)^{\alpha-1} e^{-\beta\left(\frac{x+\lambda}{\theta}\right)^{\alpha}} e^{e^{-\beta\left(\frac{x+\lambda}{\theta}\right)^{\alpha}}}\right]$$
$$log\left[\frac{\alpha\beta}{\theta(e-1)}\left(\frac{x+\lambda}{\theta}\right)^{\alpha-1} e^{-\beta\left(\frac{x+\lambda}{\theta}\right)^{\alpha}} e^{e^{-\beta\left(\frac{x+\lambda}{\theta}\right)^{\alpha}}}\right]dx,$$

$$SE_x = -\frac{\alpha\beta}{\theta(e-1)}log\left[\frac{\alpha\beta}{\theta(e-1)}\right]\int_{-\lambda}^{\infty}\left(\frac{x+\lambda}{\theta}\right)^{\alpha-1} e^{-\beta\left(\frac{x+\lambda}{\theta}\right)^{\alpha}} e^{e^{-\beta\left(\frac{x+\lambda}{\theta}\right)^{\alpha}}}dx, \tag{1}$$

$$-\frac{\alpha\beta(\alpha-1)}{\theta(e-1)}\int_{-\lambda}^{\infty}\left(\frac{x+\lambda}{\theta}\right)^{\alpha-1} log\left(\frac{x+\lambda}{\theta}\right) e^{-\beta\left(\frac{x+\lambda}{\theta}\right)^{\alpha}} e^{e^{-\beta\left(\frac{x+\lambda}{\theta}\right)^{\alpha}}}dx, \tag{2}$$

$$+\frac{\alpha\beta^2}{\theta(e-1)}\int_{-\lambda}^{\infty}\left(\frac{x+\lambda}{\theta}\right)^{2\alpha-1} e^{-\beta\left(\frac{x+\lambda}{\theta}\right)^{\alpha}} e^{e^{-\beta\left(\frac{x+\lambda}{\theta}\right)^{\alpha}}}dx, \tag{3}$$

$$-\frac{\alpha\beta}{\theta(e-1)}\int_{-\lambda}^{\infty}\left(\frac{x+\lambda}{\theta}\right)^{\alpha-1} e^{-2\beta\left(\frac{x+\lambda}{\theta}\right)^{\alpha}} e^{e^{-\beta\left(\frac{x+\lambda}{\theta}\right)^{\alpha}}}dx. \tag{4}$$

Substituting $w = \beta\left(\frac{x+\lambda}{\theta}\right)^{\alpha}$ and solving Eqs. (1), (2), (3), and (4) separately.
Eq. (1) is then equal to $-log\left(\frac{\alpha\beta}{\theta(e-1)}\right)$

In addition, Eq. (5) using the expansions (9),(18) and (37) can be expressed as follows:

$$-(\alpha-1)log\left(\frac{\lambda}{\theta}\right) - \frac{(\alpha-1)}{(e-1)}\sum_{i=0}^{\infty}\sum_{n=0}^{\infty}\sum_{k=0}^{i}\frac{(-1)^{k+1}}{(n!)i}\binom{i}{k}\frac{\theta^k\Gamma(\frac{k}{\alpha}+1)}{\lambda^k\beta^{\frac{k}{\alpha}}(n+1)^{\frac{k}{\alpha}+1}}$$

Moreover, using the series (9), Eq. (3) is expressed as follows:

$$\frac{1}{e-1}\sum_{n=0}^{\infty}\frac{1}{(n+1)!(n+1)},$$

Finally, Eq. (4) is equal to $\frac{1}{1-e}$.
Therefore, the Shannon entropy of the LKME distribution is expressed as follows:

$$SE_x = \frac{1}{1-e}\sum_{n=0}^{\infty}\sum_{i=0}^{\infty}\sum_{k=0}^{i}\frac{1}{(n+1)!}\left[\binom{i}{k}\frac{(-1)^{k+1}}{i}\frac{(\alpha-1)\theta^k}{\lambda^k\beta^{\frac{k}{\alpha}}}\frac{\Gamma(\frac{k}{\alpha}+1)}{(n+1)^{\frac{k}{\alpha}}} - \frac{1}{n+1}\right]$$
$$+\frac{1}{1-e} - \left[log\left(\frac{\alpha\beta}{\theta(e-1)}\right) + (\alpha-1)log\left(\frac{\lambda}{\theta}\right)\right].$$
